# DNA methylation in the adipose tissue and whole blood of Agent Orange-exposed Operation Ranch Hand veterans: a pilot study

**DOI:** 10.1186/s12940-021-00717-y

**Published:** 2021-04-13

**Authors:** Matthew R. Rytel, Rondi Butler, Melissa Eliot, Joseph M. Braun, E. Andres Houseman, Karl T. Kelsey

**Affiliations:** 1 Department of Epidemiology, Brown University School of Public Health, Providence, RI 02912 USA; 2 Department of Pathology and Laboratory Medicine, Brown University School of Public Health, Providence, RI 02912 USA; 3 Statistical Bioinformatics, GlaxoSmithKline, 1250 S Collegeville Rd, Collegeville, PA 19426 USA

**Keywords:** Adipose, Blood, Dioxin, Agent Orange, DNA methylation, Epigenetics

## Abstract

**Background:**

Between 1962 and 1971, the US Air Force sprayed Agent Orange across Vietnam, exposing many soldiers to this dioxin-containing herbicide. Several negative health outcomes have been linked to Agent Orange exposure, but data is lacking on the effects this chemical has on the genome. Therefore, we sought to characterize the impact of Agent Orange exposure on DNA methylation in the whole blood and adipose tissue of veterans enrolled in the Air Force Health Study (AFHS).

**Methods:**

We received adipose tissue (*n* = 37) and whole blood (*n* = 42) from veterans in the AFHS. Study participants were grouped as having low, moderate, or high TCDD body burden based on their previously measured serum levels of dioxin. DNA methylation was assessed using the Illumina 450 K platform.

**Results:**

Epigenome-wide analysis indicated that there were no FDR-significantly methylated CpGs in either tissue with TCDD burden. However, 3 CpGs in the adipose tissue (contained within *SLC9A3*, *LYNX1*, and *TNRC18*) were marginally significantly (*q* < 0.1) hypomethylated, and 1 CpG in whole blood (contained within *PTPRN2*) was marginally significantly (*q* < 0.1) hypermethylated with high TCDD burden. Analysis for differentially methylated DNA regions yielded *SLC9A3*, among other regions in adipose tissue, to be significantly differentially methylated with higher TCDD burden. Comparing whole blood data to a study of dioxin exposed adults from Alabama identified a CpG within the gene *SMO* that was hypomethylated with dioxin exposure in both studies.

**Conclusion:**

We found limited evidence of dioxin associated DNA methylation in adipose tissue and whole blood in this pilot study of Vietnam War veterans. Nevertheless, loci in the genes of *SLC9A3* in adipose tissue, and *PTPRN2* and *SMO* in whole blood, should be included in future exposure analyses.

## Background

Operation Ranch Hand (1962–1971) was a mission conducted by the US military during the War in Vietnam in which the Air Force dispersed millions of gallons of herbicides across Vietnam to destroy vegetative cover and crops that benefitted enemy forces [1]. The most widely used herbicide was Agent Orange, and the soldiers assigned to Operation Ranch Hand were exposed to it during mixing, loading, spraying, and clean-up duties [1]. The defoliant was contaminated with 2,3,7,8-tetrachlorodibenzo-*p*-dioxin (TCDD), which was a byproduct of the manufacturing process. TCDD is lipophilic and bioaccumulates, persisting in the human body with a half-life of 7 to 11 years [1]. TCDD is highly toxic and has been shown to disrupt endocrine signaling, trigger oxidative stress, and alter the epigenome [2, 3].

Shortly after the end of Operation Ranch Hand, the veterans reported health issues that they believed were related to their Agent Orange exposure [4]. This prompted the Air Force to launch a prospective, longitudinal study in 1982 termed the Air Force Health Study (AFHS), which was designed to track morbidity of the Ranch Hand veterans [4]. Using data from the AFHS, studies have tied a variety of conditions to Agent Orange exposure. The National Academies of Sciences has concluded that there is “sufficient evidence of an association” of Agent Orange exposure with soft-tissue sarcoma, non-Hodgkin lymphoma, chronic lymphocytic leukemia, Hodgkin lymphoma, chloracne, hypertension, and monoclonal gammopathy of undetermined significance [1]. However, the impact of Agent Orange exposure on the epigenome of the Ranch Hand veterans “remains uncertain” [1]. Therefore, targeting our analysis on methylation in the DNA of adipose and whole blood tissue samples collected as part of the AFHS, we sought to characterize the effect of TCDD exposure on the epigenome of the Ranch Hand veterans.

Adipose tissue plays a key regulatory role in fatty acid and cholesterol metabolism, which when dysregulated, can contribute to cardiovascular disease [5, 6]. Also, adipose tissue has a range of endocrine functions that influence multiple processes including insulin signaling, appetite, blood pressure, and immune function [5, 6]. TCDD has previously been shown to both augment and inhibit lipid accumulation in adipocytes [7–11]. Further, murine adipocyte differentiation was inhibited by TCDD exposure in vitro, and this change was accompanied by global demethylation [12]. TCDD also hampers insulin-mediated glucose uptake in 3 T3-L1 adipocytes, possibly linking exposure to type II diabetes [13]. Gene expression analyses in human adipocytes and preadipocytes provide evidence that TCDD impacts metabolic pathways, and also upregulates genes related to the inflammatory immune response and cancer [14]. Taken together, gene expression and regulation in adipose tissue has a variety of impacts on health, and these processes clearly could be influenced by exposure to TCDD.

Methylation in whole blood has been examined as a biomarker for a variety of pathologies including cancer, metabolic disorders, and cardiovascular disease (reviewed in [15]). Observing changes in methylation in the whole blood of adults living in Anniston, Alabama who were exposed to the organochlorine class termed polychlorinated biphenyls (PCBs), including TCDD, Pittman et al. found 28 CpGs and 115 differentially methylated regions (DMRs) to be significantly aberrantly methylated with elevated serum levels of PCBs [16]. In a subsequent follow-up of the same study, 116 CpGs were significantly associated with PCB burden at the 5% FDR level [17]. Studies on these Anniston adults have drawn associations between PCB exposure and hypertension, liver disease, metabolic syndrome, elevated serum lipid levels, and diabetes [16]– effects thought to be associated with exposure to these chemicals. Alterations to the epigenome of whole blood in adults has been observed in other instances of exposure to persistent polychlorinated organic pollutants, as well [18–21]. Here, we conducted a pilot EWAS of both peripheral blood and adipose tissue, examining the association of DNA methylation changes in these tissues with measured TCDD levels in Ranch Hand AFHS participants.

## Methods

Potential participants of the AFHS were identified via an in-depth search of all organizational records stored at the Military Records Division, National Personnel Records Center, St. Louis, Missouri [22]. All Ranch Hand veterans were targeted as the study population and 99.5% were located [23]. Contacted Ranch Hand veterans and control participants were fully informed of the nature and purpose of the medical examinations and tests and signed an informed consent form before enrolling in the AFHS [22]. Consenting Operation Ranch Hand veterans and their matched controls underwent physical examinations at time points 1, 3, 5, 10, 15, and 20 years after the initiation of the Air Force Health Study in 1982 [24]. The control participants were a similar group of soldiers who flew cargo aircraft elsewhere in Southeast Asia between 1962 and 1971 and were not exposed to Agent Orange or other herbicides [24]. The Center for Disease Control and Prevention completed additional blood draws in 777 Ranch Hand veterans and 1174 control participants at 5, 10, and 15 years after the initiation of the AFHS and measured serum TCDD levels [24]. To study methylation features associated with dioxin exposure, we stratified participants by lipid-adjusted serum dioxin (TCDD) levels into four TCDD burden groups: control, low, moderate, and high.

Whole venous blood samples were collected from Ranch Hand and control veterans in cycles 1 (1982) and 6 (2002) respectively [25]. Adipose tissue samples were extracted from a subset of participants in cycle 5 (1997) [24, 25]. The protocol used to isolate and process whole blood samples was described in the Vietnam Experience Study [26]. Adipose tissue collection and processing methods were also described in the Science Applications International Corporation test plans for sample collection [25, 27].

We received whole blood and adipose samples and isolated genomic DNA. Briefly, we digested 6.5–45.4 mg of adipose tissue in Qiagen Buffer ATL and proteinase K at 56 °C for 2 h with agitation (300 rpm in an Eppendorf Thermomixer; occasional gentle vortexing to disrupt tissue). Then, we treated DNA with 150 U/ml RNase A for 10 min at room temperature and subsequently recovered it using the Zymo Clean & Concentrator-5 kit as directed. Employing the Qiagen QIAmp DNA Blood Mini Kit *per* manufacturer instructions, we extracted whole blood DNA. We bisulfite converted all DNA samples using the Zymo EZ DNA Methylation kit and applied the alternative incubation conditions for Illumina Infinium Methylation Array samples. We sent samples to the UCSF Genomics Core Facility, Institute for Human Genetics, for array processing.

We used the Illumina 450 K Infinium Methylation platform to measure global DNA methylation levels in the Ranch Hand veteran and control groups. Processed and normalized data met all quality control standards. On the Infinium 450 K platform, DNA methylation is typically measured on a “beta” scale, representing the fraction of methylated molecules (for each target site), bounded by 0 (unmethylated) and 1 (methylated).

We completed all statistical analyses using R statistical software [28]. Upon obtaining average beta values from the Infinium 450 K array we normalized the data and executed quality assurance and quality control measures. This included removal of poor quality CpGs and samples on the basis of detection *p*-value [29], discovery and removal of cross reactive CpGs [30], and the removal of CpGs containing polymorphic sites with a minor allele frequency > 1% at the target site or within the probe sequence. We corrected technical variation in specimen sequencing associated with non-specific fluorescence using the Noob method [31] of the Bioconductor package *minfi*. Next, we utilized the FunNorm procedure [32] for functional normalization to correct for between-array variance associated with control probes. Employing the BMIQ technique [33], we then corrected for variance associated with the two different probe designs present in the array. Finally, we adjusted for batch effects using the ComBat method [34]. After these steps, the original number of CpGs was lowered from 485,577 to 485,283 loci for adipose tissue, and to 485,211 for whole blood.

We used an epigenome wide association study (EWAS) to investigate the relationship between dioxin burden and methylation at individual CpG loci across the entire adipose tissue genome. We examined potential confounding variables using AFHS participant data including smoking history, body mass index (BMI), and age. To estimate the number of additional confounders, we conducted a surrogate variable analysis (SVA) [35] with the Bioconductor SVA package, finding five surrogate variables. Next, we employed linear models with empirical Bayes estimation to test for a possible association between dioxin levels and differential methylation. We included smoking history (ever or never), BMI (kg/m^2^), age (years), and the surrogate variables in the model as covariates. We modeled dioxin level as a categorical variable (e.g., control vs. high). Finally, we used the Benjamini-Hochberg (BH) method to control the false discovery rate (FDR). We ordered CpGs by BH-adjusted *p*-values in an increasing fashion to identify significantly differentially methylated loci.

We also examined regional changes in DNA methylation applying the DMRcate package (Bioconductor), which has been shown to outperform other tools used to study differentially methylated regions of DNA [36]. Excluding CpGs located within SNPs, DMRcate identified changes in beta values for each of the 46,470 regions in the control and high serum TCDD groups. We measured significance using the Stouffer *p*-value (regional level), and the minimum adjusted *p*-value from the CpGs within the regions. Regions were ordered according to the minimum adjusted *p*-value to identify DMRs potentially differentially methylated in the high burden group compared to the control.

We proceeded to analyze the whole blood data, executing an EWAS that used SVA to account for white blood cell composition and any additional confounders. To compare methylation levels in the control versus high burden groups, we built linear models with empirical Bayes estimation that adjusted for age (years), BMI (kg/m^2^), smoking history (ever vs. never), and the 8 surrogate variables identified using SVA. Again, we employed BH-method to control the FDR. Following this EWAS using SVA, we deconvoluted the blood data to obtain estimates of the proportions of the different leukocytes in the sample. The neutrophil to lymphocyte and lymphocyte to monocyte ratios (NLR and LMR respectively) were calculated for each participant. Subsequently, we carried out additional genome-wide scans comparing methylation betas in the control and high dioxin load groups, this time adjusting for the demographic data and the blood cell proportions or the demographic data and the NLR. After employing the BH method to control the FDR, we ordered CpGs according to their adjusted *p*-value in order to identify significantly differentially methylated loci. In line with our procedures from the previous sample types, we applied DMRcate to identify regions of differentially methylated DNA with elevated serum TCDD levels.

Abnormal leukocyte proportions, and in particular, the NLR and the LMR are indices of systemic inflammation and can serve as predictors for diseases like cancer [37, 38]. We used t-tests to compare cell type proportions, the NLR, and the LMR between the highly exposed group and the control group.

Using data from the Anniston Community Health Survey (ACHS) II [17] we performed a candidate-CpG analysis with the whole blood data. We identified the 116 significantly methylated CpG loci with dioxin exposure at the 5% FDR level in the AFHS data [17], and subsequently, performed t-tests comparing methylation levels in the high burden group compared to the controls. Another study targeted CYP1A1 and IGF2 in a candidate-gene analysis of the whole blood of Vietnamese individuals potentially exposed to Agent Orange [39]. At each of the CpGs within CYP1A1 and IGF2 in the AFHS whole blood 450 K data, we executed a t-test to compare methylation levels between the highly burdened group and the controls. Finally, we performed a candidate analysis using the same approach in both the adipose tissue and whole blood data for genes within the AHR pathway (AHR, AHRR, CYP1A1, CYP1A2, CYP1B1, and ARNT).

We next used the Hannum, Horvath, and phenoAge epigenetic clock analyses to estimate the chronological ages of the Ranch Hand veterans and controls in whole blood. The Hannum clock is based on the methylation profile at 71 CpGs and was constructed using, and tested on, whole blood data [40, 41]. This clock also takes into account age-related shifts in blood cell proportions [40, 41]. In contrast, the Horvath clock was built using methylation data from multiple tissues, and includes a set of 353 CpGs [41, 42]. PhenoAge consists of a set of 513 CpGs, and was trained using whole blood methylation data and on chronological age as well as aging- and lifestyle-related disease phenotypes [43]. PhenoAge has been shown to perform with high precision in tissues other than whole blood [43]. We also estimated chronological age using the adipose tissue data. However, due to the fact that the Hannum clock is best suited for use with whole blood data, we did not apply this method to the adipose tissue data.

We completed the Hannum and Horvath clock analyses through the wateRmelon package; the PhenoAge clock was accessed through the ENmix package. For each participant, we calculated age acceleration by regressing methylation age on chronological age and taking the residuals from the model [44]. We examined possible associations between age acceleration and dioxin burden using linear models. In this analysis, we modeled serum dioxin levels both as categorical and continuous variables. For analysis with the whole blood data, we constructed five different models, which accounted for covariates that could impact methylation age estimates, to assess the relationship between age acceleration and DNA methylation: adjusting for cell type proportions (model 1), NLR (model 2), BMI, and smoking history (model 3), cell type proportions, BMI, and smoking history (model 4), and NLR, BMI, and smoking history (model 5). Only model 3 was applied to the adipose tissue data. The cell type proportions used were the estimates generated from the deconvolution analysis.

## Results

In the adipose tissue data, we stratified participants (exposed and control veterans) based on lipid-adjusted serum dioxin levels (LSDL) into four burden groups: control (LSDL ranging from 2.21 to 7.05 ppt; *n* = 12), low (LSDL ranging from 5.6 to 7.24 ppt; *n* = 3), moderate (LSDL between 8.9 and 19.6 ppt; *n* = 11), and high (LSDL ranging from 26.6 to 167.6 ppt; *n* = 11) (Table 1). We grouped participants in the same manner in the whole blood data: control (LSDL ranging from 2.21 to 7.05 ppt; *n* = 11), low (LSDL ranging from 5.6 to 7.24 ppt; *n* = 4), moderate (LSDL between 8.9 and 19.6 ppt; *n* = 11), and high (LSDL between 26.6 and 167.6 ppt; *n* = 15). There were no participants with non-detectable dioxin levels in either tissue group. Serum dioxin concentration was the only variable that significantly differed between the control and high TCDD burden groups.

**Table 1:** Demographic data in each burden group in the adipose and whole blood tissue categories

**Adipose**	**Control (*N*** **= 12)**	**Low (*N*** **= 3)**	**Medium (*N*** **= 11)**	**High (*N*** **= 11)**	**All (*N*** **= 37)**
Age [Mean (SD)] (yrs)	70.5 (6.5)	81.7 (11.6)	77.0 (8.5)	67.7 (1.9)	72.5 (7.9)
Smoking: ever [N (%)]	9 (75.0)	2 (66.7)	8 (72.7)	8 (72.7)	27 (73.0)
BMI [Mean (SD)] (kg/m^2)	32.1 (5.5)	24.9 (1.0)	30.9 (3.7)	30.9 (3.3)	30.8 (4.4)
Dioxin [Mean (SD), Range] (ppt)	4.2 (1.3), 2.2–7.1	6.7 (0.9), 5.6–7.2	13.4 (3.3), 8.9–19.6	66.4 (48.6), 26.6–167.6	25.6 (37.4), 2.2–167.6
**Whole Blood**	**Control (*N*** **= 12)**	**Low (*N*** **= 4)**	**Medium (*N*** **= 11)**	**High (*N*** **= 15)**	**All (*N*** **= 42)**
Age [Mean (SD)] (yrs)	70.6 (6.5)	79.5 (10.4)	77.0 (8.5)	69.7 (5.8)	72.8 (7.9)
Smoking: ever [N (%)]	9 (75.0)	3 (75.0)	8 (72.7)	11 (73.3)	31 (73.8)
BMI [Mean (SD)] (kg/m^2)	32.1 (5.5)	25.7 (1.8)	30.9 (3.7)	29.7 (3.5)	30.3 (4.4)
Dioxin [Mean (SD), Range] (ppt)	4.2 (1.3), 2.2–7.1	6.5 (0.8), 5.6–7.2	13.4 (3.3), 8.9–19.6	60.4 (42.3), 26.6–167.6	26.9 (35.6), 2.21–167.6

Demographic data from the Ranch Hand and control veterans was gathered as part of the Air Force Health Study. The burden groups are stratified by lipid-adjusted serum dioxin levels

We began epigenomic analysis of the adipose tissue DNA with an EWAS to determine if any individual loci were aberrantly methylated to a level of genome-wide significance in the highly burdened Ranch Hands compared with the control veterans. We found 35,216 CpGs to be significantly differentially methylated in the high dioxin load group compared to the control (*p* < 0.05); however, no CpGs survived correction for multiple comparisons at any loci. Three CpGs (cg18447419, cg04350571, and cg04939944) showed marginal genome-wide significant methylation (q < =0.1) (Table 2). These marginally significant loci were all hypomethylated and were associated with the genes *SLC9A3*, *LYNX1*, and *TNRC18*. Additionally, *LOC14937* contained two of the 50 most significantly altered CpGs by *q*-value, and in each case, the locus was hypomethylated as indicated by the associated regression coefficient (Table 2). Similarly, *STAB1* contained two of the CpGs within the 50 lowest *q*-value group, and in each case the loci were hypermethylated (Table 2). Furthermore, since dioxin is known to interact with genes in the AHR pathway, members of this pathway were examined for TCDD serum associated methylation alterations, but no statistically significant relationships were found (data not shown).

**Table 2:** Top 50 methylated adipose loci in the highly burdened veterans, ordered by *q*-value

**CpG**	**Chromosome**	**Gene**	**Regression Coeff.**	**pval**	**qval**
cg18447419	5	SLC9A3	−0.0951	1.07E-07	0.052
cg04350571	8	LYNX1	−0.0201	5.97E-07	0.100
cg04939944	7	TNRC18	−0.0302	6.20E-07	0.100
cg26351451	4	KIAA1239	0.0364	1.10E-06	0.134
cg10831901	20	LOC149837	−0.0773	1.44E-06	0.140
cg07327335	2	PP14571,GPC1	−0.0466	1.91E-06	0.155
cg09130535	11	ADRBK1	−0.0197	3.11E-06	0.213
cg14633910	6	SYCP2L	−0.0253	4.40E-06	0.213
cg25679766	6		0.0315	4.43E-06	0.213
cg18584639	5		0.0346	4.54E-06	0.213
cg23045716	4	LOC100130872,LOC100130872-SPON2	0.0227	5.10E-06	0.213
cg21726840	19	PRAM1	0.0237	5.26E-06	0.213
cg26132511	6	ZFAND3	0.0231	8.33E-06	0.292
cg12520727	11	CLPB	−0.0226	8.78E-06	0.292
cg01026256	3	VWA5B2,MIR1224	−0.0441	9.11E-06	0.292
cg13983779	9		−0.0245	9.63E-06	0.292
cg06647068	12	CHST11	−0.0341	1.14E-05	0.314
cg10422455	11	MRGPRX2	0.0252	1.16E-05	0.314
cg05361530	16		0.0217	1.31E-05	0.320
cg07392385	10	SORCS1	−0.0327	1.34E-05	0.320
cg13689342	21		0.0202	1.42E-05	0.320
cg09977640	17		−0.0291	1.55E-05	0.320
cg13905238	3		−0.0310	1.55E-05	0.320
cg05060534	13	TFDP1	−0.0140	1.61E-05	0.320
cg16156617	6	SLC17A4	0.0212	1.67E-05	0.320
cg09897374	6		0.0364	1.72E-05	0.320
cg17094014	6	MDFI	−0.0219	1.88E-05	0.338
cg07325692	7		0.0268	2.08E-05	0.351
cg25195158	12		−0.0274	2.15E-05	0.351
cg18099408	3	STAB1	0.0304	2.22E-05	0.351
cg20266320	1		−0.0330	2.31E-05	0.351
cg26284544	19	SNAPC2	−0.0678	2.38E-05	0.351
cg04307083	4	HOPX	0.0190	2.38E-05	0.351
cg09167779	5	MCC	0.0200	2.48E-05	0.354
cg13731106	9		0.0257	2.78E-05	0.358
cg21207003	3	QTRTD1	−0.0198	2.82E-05	0.358
cg10891482	11	MS4A8B	0.0226	3.01E-05	0.358
cg01540102	16	DOK4	−0.0285	3.03E-05	0.358
cg11404049	1	CASP9	−0.0141	3.10E-05	0.358
cg08644276	2		0.0265	3.12E-05	0.358
cg16763089	20	LOC149837	−0.0868	3.17E-05	0.358
cg15791105	12	COL2A1	0.0159	3.18E-05	0.358
cg25965864	12	LRRC23	−0.0235	3.25E-05	0.358
cg07860645	6	DAAM2	−0.0234	3.37E-05	0.358
cg16673904	17	SHBG	0.0199	3.39E-05	0.358
cg23477257	7	PHTF2	0.0214	3.39E-05	0.358
cg05053979	14	FOXN3	−0.0352	3.59E-05	0.365
cg01330280	6	HIST1H3H,HIST1H2BL	−0.0547	3.65E-05	0.365
cg15147215	3	STAB1	0.0218	3.83E-05	0.365
cg09357935	11	SLC6A5	−0.0453	3.85E-05	0.365

Data generated from a linear model with empirical Bayes estimation, which we used for the adipose tissue DNA methylation beta value comparisons between the Ranch Hand veterans with high serum TCDD levels (*n* = 11) and the control group (*n* = 12). The *q*-value was calculated using the Benjamini-Hochberg method. The regression coefficient identifies the direction of methylation change between the burden groups and was determined via the model we constructed. Genes associated with each CpG are also given in the table

After adjusting for multiple testing, none of the 46,470 DMRs studied were altered to reach a significant Stouffer *p*-value in the adipose tissue data. However, comparing the high to control burden groups, 11 of the regions resulted in a minimum adjusted *p*-value < 10^− 6^ based on methylation at the CpGs contained within the regions (Table 3). Many of these DMRs contain genes associated with non-coding RNAs, such as long non-coding RNAs and small nucleolar RNAs. A DMR containing *SLC9A3* had a minimum adjusted *p*-value < 10^− 6^ and was demethylated, as was the marginally significant individual locus identified within the same gene. All significant DMRs, except for one, were demethylated in the high burden group compared to the control (Table 3).

**Table 3:** Regions of adipose tissue DNA altered in the high burden group compared to the control that resulted in a minimum adjusted *p*-value < 10^− 6^

**Chrm.**	**Number of CpGs**	**pval**	**Stouffer-adjusted pval**	**Max beta-fold change**	**Mean beta-fold change**	**Overlapping Genes**
2	9	8.26E-15	1	−0.077	− 0.057	SNORA64, SNORA12, SNORA74, SNORA19, PAX8-AS1, snR65, 5S_rRNA, SNORA4, SNORD11, SNORD51, SNORA41, SCARNA6, SNORD39, SNORA75, ACA59, PAX8, SNORA48, SNORD56, SNORA43, SNORA1, Vault
13	5	8.32E-13	1	−0.046	−0.038	SNORD36
1	8	7.11E-11	1	−0.058	−0.027	snoU13, Y_RNA, SCARNA16, U1, SCARNA17, SCARNA18, SCARNA24, SNORD112, SNORA62, SNORA63, C1orf50, SNORD46, SNORA2, SNORD81, U3, SNORA51, SNORA25, SCARNA20, SNORA67, U6, SNORA70, SNORA77, SNORA26, U8, SCARNA11, SNORA31, SNORA42, SNORA40, SNORD64, ACA64, snoU109, SNORD60, SNORD116
20	9	7.97E-11	1	−0.076	−0.053	LINC00654
6	21	3.67E-09	1	−0.050	−0.024	SNORA38, LY6G5C, SNORA20
19	25	1.76E-08	1	−0.035	−0.011	USP29
1	5	1.83E-08	1	−0.058	− 0.044	snoU13, Y_RNA, SCARNA16, SCARNA21, U1, SCARNA17, SCARNA18, SCARNA24, SNORD112, SNORA62, SNORA63, SNORD46, SNORA2, SNORD81, U3, SNORA51, SNORA25, SCARNA20, SNORA67, U6, SNORA70, SNORA77, SNORA26, U8, SCARNA11, SNORA31, SNORA42, SNORA40, SNORD64, ACA64, snoU109, SNORD60
5	12	2.98E-08	1	−0.068	−0.014	SLC9A3
17	12	6.86E-08	1	−0.058	−0.025	C17orf107, CHRNE
7	16	9.88E-08	1	0.071	0.013	YWHAG
11	10	1.61E-07	1	−0.042	−0.030	LSP1, SLC6A5

Data generated using the DMRcate algorithm from Bioconductor. The “pval” is a minimum adjusted *p*-value generated by the DMRcate algorithm, and we used a significance cutoff of 1e-6 when comparing methylation across the adipose tissue DNA regions in the Ranch Hand veterans with high serum TCDD (*n* = 11) levels compared to the control group (*n* = 12). The Stouffer-adjusted *p*-value is also calculated by DMRcate. The max and mean beta-fold change indicate differences in methylation levels between the highly burdened and control groups. The “Overlapping Genes” column indicates the genes associated with the region of DNA studied

An EWAS conducted with the whole blood data did not reveal any significantly methylated loci at the 5% FDR level. However, one CpG (cg13442689), which was associated with the gene *PTPRN2*, was marginally significantly hypermethylated (*q* < =0.1). Closer evaluation of the 50 lowest *q*-values (Table 4) revealed genes containing multiple loci. Two of the top 50 significantly methylated whole blood CpGs were located within *MEST*, *MESTIT1* and were both more heavily methylated in the high burden group compared to the control. Also, *RASA3* held two more of these top 50 significantly methylated whole blood CpGs, and in each case they were hypomethylated in the high serum TCDD group compared to the controls.

**Table 4:** The top 50 differentially methylated CpGs in whole blood, ordered by *q*-value, when comparing the high and control burden groups

**CpG**	**CHR**	**Gene**	**Regression Coeff.**	**pval**	**qval**
cg13442689	7	PTPRN2	0.037	2.1E-07	0.100
cg04344875	7	MEST,MESTIT1	0.019	8.3E-07	0.179
cg06353830	1		−0.024	1.3E-06	0.179
cg07064673	11	HCCA2	0.016	1.5E-06	0.179
cg02500300	4	STOX2	0.014	2.1E-06	0.204
cg19558802	15	GLDN	0.047	3.1E-06	0.248
cg23154021	12		−0.059	3.7E-06	0.259
cg14950515	4		−0.048	4.5E-06	0.273
cg24393602	6	GSTA4	0.018	6.3E-06	0.337
cg06713076	17		−0.020	7.4E-06	0.354
cg04507925	2	UNC80	−0.030	9.1E-06	0.354
cg20697519	1	SLAMF8	0.021	1.0E-05	0.354
cg03450733	X	ARX	−0.061	1.0E-05	0.354
cg12948543	14	PACS2	0.014	1.2E-05	0.354
cg08084655	17	BTBD17	−0.034	1.3E-05	0.354
cg00152946	10		−0.017	1.3E-05	0.354
cg18898267	8		−0.050	1.3E-05	0.354
cg20845970	8		−0.022	1.4E-05	0.354
cg21461300	13	RASA3	−0.038	1.5E-05	0.354
cg08721112	1		−0.086	1.5E-05	0.354
cg21355100	19	ZNF555	0.024	1.5E-05	0.354
cg10881110	1	SLC25A34	0.020	1.7E-05	0.360
cg06534800	5	DOK3	0.019	1.7E-05	0.360
cg24352317	X	LOC729609	−0.032	2.1E-05	0.425
cg24757937	14	SIX1	−0.036	2.3E-05	0.425
cg22258713	8		0.048	2.3E-05	0.425
cg24851684	6	DHX16	0.017	2.4E-05	0.425
cg00893368	13	RASA3	−0.025	2.5E-05	0.436
cg11576274	1		−0.048	2.7E-05	0.449
cg27590787	X		0.089	2.9E-05	0.476
cg26708559	7	MEST,MESTIT1	0.017	3.1E-05	0.477
cg16204066	6	ITPR3	−0.024	3.2E-05	0.477
cg06439589	X	SSR4	0.014	3.3E-05	0.477
cg12905592	12	FMNL3	0.020	3.8E-05	0.477
cg08772163	13	ARL11	0.013	4.1E-05	0.477
cg22741244	2	KLHL30	−0.027	4.1E-05	0.477
cg11646505	6		0.013	4.2E-05	0.477
cg03290530	3		0.045	4.2E-05	0.477
cg08132116	3	NICN1	0.015	4.2E-05	0.477
cg21032058	14	TMEM63C	0.012	4.3E-05	0.477
cg00329180	16	ANKRD11	0.020	4.4E-05	0.477
cg00044796	1		−0.105	4.7E-05	0.477
cg13036944	3		0.013	4.7E-05	0.477
cg02184697	16	KREMEN2	−0.050	4.8E-05	0.477
cg05825400	17	ABR	0.030	5.0E-05	0.477
cg26114595	17	EFCAB5	0.019	5.0E-05	0.477
cg01987515	2	COPS7B	0.010	5.2E-05	0.477
cg17733084	1	NEXN	−0.014	5.3E-05	0.477
cg22671717	1		−0.103	5.4E-05	0.477
cg26289712	8		0.016	5.4E-05	0.477

Data generated from a linear model with empirical Bayes estimation, which we used for the whole blood DNA methylation beta value comparisons between the Ranch Hand veterans with high serum TCDD levels (*n* = 15) and the control group (*n* = 12). The *q*-value was calculated using the Benjamini-Hochberg method. The regression coefficient identifies the direction of methylation change between the burden groups and was determined via the model we constructed. Genes associated with each CpG are also given in the table

Our epigenome-wide analysis that adjusted for estimated leukocyte proportions in the whole blood data revealed no significantly methylated loci after accounting for multiple testing. Additionally, neither the proportions of cell types, the NLR, nor the LMR, significantly differed between the high dioxin burden group and the controls.

With low power to find associations between serum TCDD levels and methylation at individual loci at the genome-wide level, we used a candidate-CpG approach to compare the Ranch Hand data with that from the ACHS II [17]. Pittman et al. (2020) reported 116 CpG loci were significantly associated with dioxin exposure at the 5% FDR level. Comparisons between the burden groups at each of these loci in the Ranch Hand data revealed one locus – cg20489453 – with significantly higher methylation levels (*p* < 0.05) associated with elevated serum TCDD concentrations. Four other CpGs were marginally significantly differentially methylated (*p* < 0.1) in the high burden versus control groups. Notably, four of the candidate CpGs were contained within the *ALLC* gene and were all hypomethylated in both the Ranch Hand and ACHS data. One of these loci located within *ALLC* (cg00999904) was marginally significantly demethylated in the high burden Ranch Hand veterans compared to the control group. In the ACHS, this locus in the *ALLC* gene had the greatest change in methylation between the exposed and control groups, and was demethylated [17].

We carried out additional analysis with the whole blood data for differentially methylated loci within the genes *IGF2* and *CYP1A1*, which were targeted in a study of the peripheral blood of a group of people living in areas of Vietnam which were sprayed with Agent Orange [39]. Giuliani et al., 2018 provide evidence that loci within *CYP1A1*, but not *IGF2*, were altered with Agent Orange exposure. We found significant differences (*p* < 0.05) in methylation at multiple loci (cg00353139 and cg19817399) in the *CYP1A1* gene in the high burden Ranch Hand veterans compared to the controls, although these differences were not systematic (i.e. methylation was not in the same direction between the two studies) and were consistent with the significance being the result of multiple comparisons. Similarly, though the loci (cg01351425 and cg00221747) we found to be significantly differently methylated (*p* < 0.05) in *IGF2* in the highly burdened veterans compared to the control were both hypomethylated, these results were also consistent with arising as the result of multiple comparisons. In addition to *CYP1A1*, we explored other AHR pathway genes for differential methylation associated with serum dioxin levels. Ultimately, differences in methylation between the high and control dioxin groups in the genes of the AHR pathway were not significant when accounting for multiple comparisons (data not shown).

None of the DNA regions included in our DMRcate analysis in the whole blood data were significantly methylated between the high and control groups, by either the Stouffer-adjusted or minimum adjusted *p*-values.

There was no significant difference (t-test) in methylation age values using the Hannum, Horvath, and PhenoAge epigenetic clocks between the high serum TCDD and control groups in the whole blood data. Additionally, there was no correlation between methylation age and dioxin levels (modeled as a continuous variable). For each clock, only chronological age was significantly correlated with methylation age (Hannum clock: *r* = 0.900 *p* < 0.01; Horvath clock: *r* = 0.882, *p* < 0.01; PhenoAge clock: *r* = 0.880, *p* < 0.01). Age acceleration was calculated by regressing methylation age on chronological age and taking the residuals of the model [44]. We constructed linear models with age acceleration as the outcome and dioxin level as the predictor (see methods). After adjusting for confounders, we determined that age acceleration was not associated with elevated serum dioxin levels. We repeated this analysis with the adipose tissue data (excluding the Hannum clock) and similarly observed no association of age acceleration with TCDD level, and only chronological age was significantly correlated with methylation age (Horvath: *r* = 0.855, *p* < 0.01; PhenoAge clock: *r* = 0.788, *p* < 0.01).

## Discussion

We evaluated the DNA methylation of the adipose tissue and whole blood of a pilot-sized subset of Operation Ranch Hand veterans who were exposed to Agent Orange while serving in the Vietnam War. There were no significant TCDD-associated changes in the adipose tissue methylome at a genome-wide level after correcting for multiple comparisons. However, three individual loci in the adipose tissue were marginally significantly hypomethylated with increased serum TCDD levels. These three loci were contained within the genes *SLC9A3*, *LYNX1*, and *TNRC18*.

There is limited data on the biological role of SLC9A3, however, the gene encodes a membrane Na/H exchanger 3 [45], and is associated with the RhoA GTPase signaling pathway [46]. The RhoA GTPase and its downstream targets regulate cell adherence, migration, and proliferation through actin-cytoskeletal modification, and abnormal activation of this pathway plays a role in several diseases [47, 48]. Interestingly, the aryl hydrocarbon (dioxin) receptor (AHR), which mediates the toxic effects of TCDD, interacts with the RhoA and Rac1 signaling pathways through the Vav3 exchange factor to influence cell morphology and adhesion, and positively regulates cell migration [49, 50]. While the impact of hypomethylation at cg18447419 on the action of AHR-mediated RhoA/Rac1 signaling is unknown, this CpG and *SLC9A3* should be considered in future candidate analyses given the known interaction between this pathway and dioxin and its potential role in diseases like cancer.

Data suggest that dioxin exposure may play a role in modulating adipose tissue levels in the body. *STAB1*, which we discovered could be coordinately hypermethylated in the adipose tissue of the Ranch Hands compared to the control veterans, has been shown to be overexpressed in the white adipose tissue of rats exposed to TCDD [51]. This increased expression of *STAB1* was likely induced by TCDD-related cell death in the adipose tissue, and the authors hypothesized that it could be associated with macrophage infiltration [51]. TCDD can induce fatal wasting syndrome in rats, characterized by loss of adipose and body weight, and increased serum lipid levels [7, 8, 52], and *STAB1* overexpression could be associated with this degradation of stored fat that occurs with acute exposure to high levels of TCDD [9].

Mice chronically exposed to TCDD while being fed a high-fat diet showed significant weight gain and the development of an obese phenotype [9]. In contrast to this finding in mice, BMI was inversely associated with maternal TCDD concentration in daughters exposed in utero from a factory explosion in Seveso, Italy [10]. However, there was no such relationship in the sons exposed to TCDD in utero from the Seveso accident [10]. A prospective study of a cohort of adult French women found that higher estimated chronic dietary intake of dioxin was significantly associated with decreased BMI [53]. Data suggests that *STAB1* expression levels are increased in the subcutaneous adipose tissue of obese compared to lean individuals [54]. Given that the present study found two of the top 50 altered adipose tissue CpGs to be hypermethylated in *STAB1*, this gene could have been suppressed with TCDD burden in the Ranch Hand veterans. If *STAB1* overexpression is a marker for obesity, hypermethylation in this gene could be associated with a leaner body type in the veterans, similar to that of the French women chronically exposed to TCDD. However, no differences in BMI were observed between the Ranch Hand and control veterans when they were enrolled in the AFHS. This could have been complicated by the fact that BMI was measured in the enrolled veterans at least 11 years following their period of service.

When we applied the DMRcate algorithm to the adipose tissue data, 11 out of 46,470 searched regions were differently coordinately methylated in the high serum TCDD versus control groups by a minimum adjusted *p*-value < 10^− 6^. The DMR associated with *SLC9A3* was significantly differentially methylated, suggesting that loci across the gene were aberrantly altered with elevated serum dioxin levels, in addition to the single CpG discovered in our EWAS. Most of the other genes associated with the significantly methylated regions encoded non-coding RNAs. This is similar to our prior analysis of methylation in the sperm genome of these Ranch Hand veterans, where the gene *H19*, which encodes a long non-coding RNA (lncRNA), was altered [55]. Recent studies measuring the expression profile of lncRNAs suggest that these molecules could regulate gene expression related to the cellular responses to TCDD [56, 57]. Given this evidence that methylation and expression of genes encoding lncRNAs could be altered with TCDD to impact cellular processes, these regions of the genome could serve as candidate DMRs in future studies of the epigenetic effects of TCDD exposure.

We employed a similar agnostic approach to studying methylation at individual CpGs in the whole blood of the veterans. One CpG, contained within *PTPRN2*, was marginally significantly differentially methylated in the highly burdened group compared to the control. In the sperm data from our previous analysis, *PTPRN2* also contained a CpG that was one of the top 50 altered loci by change in beta value [55], suggesting that this gene could be aberrantly methylated with dioxin exposure in multiple tissues. Intriguingly, a study that searched the human genome for dioxin response elements – locations in the DNA where AHR binds to induce transcription – found *PTPRN2* to contain more of these loci than any other gene [58]. Thus, *PTPRN2* expression could be highly regulated by AHR and influenced by TCDD exposure. Seemingly no prior studies have examined the relationship between dioxin exposure and *PTPRN2* methylation in whole blood. Still, given the evidence that the expression of *PTPRN2* is influenced by AHR and that methylation within this gene could occur with exposure in multiple tissues, it warrants attention in future studies.

We studied the differences in whole blood methylation beta values between the highly burdened Ranch Hand and control veterans at each of the 116 differentially methylated CpGs with dioxin exposure in the Anniston adults [17]. We determined that one locus was hypomethylated in the highly burdened Ranch Hands compared to the control veterans. This locus is contained within the gene *SMO*, which codes for a 7-pass transmembrane G-protein coupled signal transduction molecule that is an important component to the hedgehog signaling pathway [59]. SMO and hedgehog family proteins are also implicated in the ERK signaling pathway, which is activated with TCDD exposure to mediate certain cell processes, including TNF-α production within white blood cells, like macrophages [60, 61]. It is unclear if, and how, hypomethylation at this specific locus within *SMO* could impact dioxin action within the blood cells through these signaling pathways. Nevertheless, this finding is noteworthy since SMO could play a role in dioxin-mediated cell processes and the CpG described here was also differentially methylated in another dioxin exposure study (ACHS).

We further supported results from the ACHS through our discovery that all four of the candidate CpGs within *ALLC* were hypomethylated in both the dioxin-exposed Anniston adults [17] and Ranch Hands veterans. Pittman et al. summarize that *ALLC* is a gene in the uric acid degradation pathway, and is expressed in a variety of tissues [17]. Also, *ALLC* has been shown to be upregulated in CD34+ hematopoietic stem cells and progenitor cells after dioxin exposure [17], however the significance of this data as it pertains to the Ranch Hand veterans and the people living in Anniston is unclear and necessitates further investigation. Additionally, the CpG with the greatest change in methylation in the Anniston adults exposed to TCDD compared to the control group (cg00999904) was marginally significantly altered in the Ranch Hand veterans. This provides evidence that this CpG, which is contained within *ALLC*, is particularly sensitive to TCDD, and should be included in further candidate analyses.

Review of the 2019 Comparative Toxicogenomics Database [62] confirmed TCDD interacts with some genes whose methylation was associated with serum TCDD levels in this study (*SLC9A3*, *LYNX1*, and *TNRC18* from adipose tissue, and *PTPRN2* from whole blood). This database review supports our data, further suggesting that these genes could be sensitive to alterations with dioxin exposure.

Since the environment has been shown to alter DNA methylation age [63], we investigated a possible association between biological age acceleration and TCDD burden in the Ranch Hand veterans. Biological age acceleration, defined as a positive difference between epigenetic age and chronological age, indicates increased risk for a host of poor health outcomes including cardiovascular disease, cancer, and premature death [64–66]. Using three different epigenetic clocks, we found no association between age acceleration and dioxin burden in the Ranch Hand veterans.

This study has some limitations. First, this was a pilot study with 37–42 veterans. Thus, we were underpowered to detect epigenetic alterations at the genome-wide level. Additionally, the latency between the time of data collection in the AFHS from the original exposure could have impacted our results. Though the Ranch Hands served in Vietnam from 1962 to 1971, the AFHS did not commence until 1982 [24], and as TCDD has a half-life in humans of 7 to 11 years [1], the serum dioxin levels we obtained did not match those at exposure. Furthermore, error in the measurement tools used to find the serum TCDD concentrations also could have influenced our analysis. Finally, while we attempted to correct for confounders, residual confounding is possible.

## Conclusions

We found limited evidence of epigenetic alterations associated with elevated serum TCDD levels in multiple tissues of Operation Ranch Hand Veterans. In our analysis of the data from the adipose tissue samples, we found the gene *SLC9A3* to be of interest for further study, as it showed significant methylation both in our CpG locus-by-locus and regional-scale tests. Also, *STAB1* – a gene that is implicated in body fat content – was coordinately methylated in the adipose tissue. In the whole blood samples, we determined that a CpG within the gene *PTPRN2*, which contains numerous dioxin response elements, was methylated to marginal genome-wide significance. Finally, we replicated findings from the ACHS, obtaining evidence that a CpG within *SMO* – a gene implicated in the action of TCDD within cells [61] – was significantly hypomethylated in both the TCDD-exposed Anniston adults and Ranch Hand veterans. All in all, data from this pilot study offers a better understanding of the impact of Agent Orange exposure on the methylome of Ranch Hand veterans, identifying candidate loci for future investigation.

## Data Availability

Data from the Anniston Community Health Survey that was analyzed in this research is available in the associated publication [15]. Data generated and analyzed from the Ranch Hand Veterans is available upon request from the corresponding author.

## Abbreviations

AFHS: Air Force Health Study
TCDD: 2,3,7,8-tetrachlorodibenzo-*p*-dioxin
PCBs: Polychlorinated biphenyls
DMRs: Differentially methylated regions
EWAS: Epigenome wide association study
BMI: Body mass index
SVA: Surrogate variable analysis
BH: Benjamini-Hochberg
FDR: False discovery rate
NLR: Neutrophil to lymphocyte ration
LMR: Lymphocyte to monocyte ration
ACHS: Anniston community health survey
LSDL: Lipid-adjusted serum dioxin levels
AHR: Aryl hydrocarbon receptor
lncRNA: Long non-coding RNA
